# Long-Term Outcomes of Collagenase Clostridium Histolyticum Injection for Palmar Dupuytren’s Deformity Correction

**DOI:** 10.7759/cureus.19952

**Published:** 2021-11-27

**Authors:** Sarang Kasture, Raj Sakamuri

**Affiliations:** 1 Trauma and Orthopaedics, Liverpool University NHS Foundation Trust, Liverpool, GBR; 2 Trauma and Orthopaedics, Ysbyty Gwynedd, Bangor, GBR

**Keywords:** outcome, complication, recurrence, collagenase clostridium histolyticum, dupuytren’s

## Abstract

Introduction

The use of collagenase *Clostridium histolyticum* injection as a minimally invasive procedure for correction of Dupuytren’s deformity is well reported in the literature. We report our experience and long-term outcome of this procedure performed in a single secondary care centre.

Methods

We prospectively evaluated 143 fingers that underwent Dupuytren’s deformity correction using CCH injection. Early side effects, degree of correction, recurrence, and patient-reported outcomes were noted.

Results

Early local complications were resolved in two weeks' duration. No major complications were encountered. More than 80% achieved immediate full correction of deformity and at an average follow-up of five years, 23% of patients had a recurrence. Around 92% were very satisfied with the procedure.

Conclusion

CCH is a safe and effective minimally invasive method to achieve correction of palmar Dupuytren’s deformity and is associated with a high patient satisfaction rate.

## Introduction

Dupuytren’s disease is a progressive proliferative disorder affecting predominantly the palmar tissue of the hand, causing progressive flexion deformity of the fingers. Men with Northern European ancestry are more commonly affected. Other risk factors include advancing age, diabetes, anti-epilepsy medication, alcoholism and smoking [1]. Various genetic factors are also reported in the literature like mitochondrial mutations, the role of HLA system and Wnt signaling pathway [2-4]. In spite of advances, there is no cure for Dupuytren’s disease and correction of Dupuytren’s deformity is the mainstay of the treatment.

Historically, surgical treatment has remained the most successful modality for correction of Dupuytren’s deformities with open fasciectomy or needle fasciotomy more popularly performed. Needle fasciotomy is associated with very high recurrence rates and is not suitable for proximal interphalangeal joint contractures due to proximity to the digital neurovascular structures [5,6]. Limited fasciectomy has a lower recurrence rate compared to needle fasciotomy but carries the risk of injury to neurovascular structures, wound healing problems, skin necrosis and infection [6].

Enzymatic fasciotomy has been reported in the literature previously [7]. Collagenase *Clostridium histolyticum* (CCH) is the first minimally invasive modality for treatment of Dupuytren’s contractures approved in Europe and the USA. Early results are encouraging and the aim of this study is to discuss the long term outcomes and patient-reported outcomes of CCH in the correction of Dupuytren’s deformity correction.

## Materials and methods

This was a service evaluation project. This product was licenced for use by European Medicines Agency in March 2011 (EMEA/H/C/002048).

Study design

This is a retrospective study of prospectively collected data of all consecutive patients with Dupuytren’s contracture treated with CCH by a single surgeon at an NHS district general hospital from January 2013 to December 2016.

Inclusion and exclusion criteria

Only those with deformity of more than 30° at the metacarpophalangeal joint (MCPJ) and more than 20° at the proximal interphalangeal joints (PIPJ) with well palpable cord in one or two fingers were included in this study. Patients undergoing repeat injections, previous surgeries on the same finger and failed follow-up (those who did not attend follow-up appointments after two reminders or moved out of the area) were excluded from the study.

Data collection

All patients were evaluated for demographic data, the number of fingers involved, joints involved and extent of deformity before injection. The degree of the deformity was recorded by a senior author with the help of a goniometer. All patients received CCH injection according to the location of deformity on Day 1. The procedure involved a standard dose of CCH (Xiapex, Swedish Orphan Biovitrum AB) along the cord with a 24G needle at one or two sites depending on the number of joints involved. They were reviewed for manipulation after 24 hours. The site of injection was evaluated for local complications of CCH injection and correction was achieved with gentle stretching over five to ten seconds after administration of local anaesthesia. Immediate complications were noted and its resolution was monitored in subsequent visits. All patients were provided with a thermoplastic extension splint for night use for three months. The splint was moulded again to a correct fit at two to four weeks as local swelling settled. Skin tears were treated with serial dressings. All patients were provided supervised physiotherapy by a hand therapist. They were followed up at one, two, six, 12 weeks, six months, 12 months and yearly after. Complete correction of the deformity was defined as residual deformity of <5°. Recurrence was defined occurrence of >20° of deformity after treatment. All patients were evaluated for early complications, the extent of correction, residual deformity and recurrence rate. Besides, patient-reported outcomes were measured using QuickDASH scoring system, with a lower score indicating a better outcome.

## Results

Out of the total 139 patients, one died two years after the procedure from medical causes and 13 were lost to follow up. These were excluded from the study. This study included 125 patients with 143 fingers; 18 patients had a concurrent finger treated in the same sitting; 97 fingers had isolated metacarpophalangeal joint involvement while 46 had metacarpophalangeal joint and proximal interphalangeal joint involvement. The average age of the patients was 69.3 years. Of the patients, 109 were male with 56% right-side involvement, 45% had a family history of the disease and the average duration of the disease was 36.3 months (Table 1).

**Table 1:** Demographic data Demographic data showing average age in years, Sex distribution ration in percentage, laterality ratio in percentage, number of patients with family history and average duration of the disease in months.

Average age in years (range)	69.3 (54 – 84)
Sex, M:F (%)	87.2 : 12.8
Laterality, Right:Left (%)	56 : 44
Family history, n (%)	56/125 (44.8%)
Average duration of disease in months (range)	36.3 (9 – 75)

**Figure 1:**
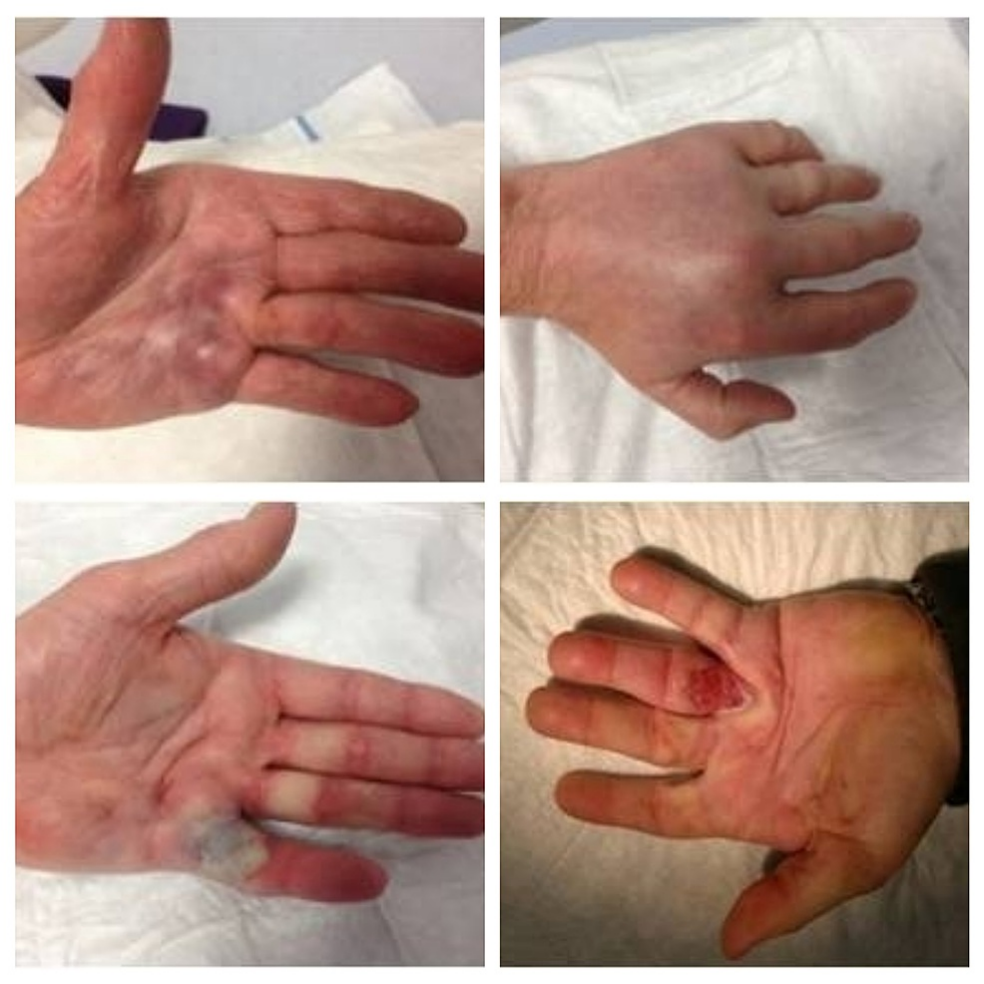
Complications of bruising, swelling, blister formation and skin tear

All patients had an immediate complication of pain, swelling and bruising of the skin after the injection; 45% had pruritis, 25% had blisters, 20% regional lymph node enlargement and 17% skin tears. No patient had tendon or ligament tears (Table 2) (Figure 1).

**Table 2:** Complications Number of patient reporting complications of pain, swelling, bruising, pruritis, blisters, regional lymphadenopathy, skin tears and ligament/tendon injury.

Complication	n (%)
Pain, swelling, bruising	143/143 (100%)
Pruritis	64/143 (44.75%)
Blisters	36/143 (25.17%)
Regional lymphnodes enlargement	29/143 (20.27%)
Skin tears	24/143 (16.78%)
Tendon/ligament injury	0/143 (0%)

The average initial deformity was 43.6° (36.5° MCPJ and 48.2° for PIPJ) which was corrected to an average 4.6° after manipulation. Around 88.1% had full correction of the deformity at the (MCPJ) and 76% at the proximal interphalangeal joint (PIPJ). Of the residual deformity, 67% was in PIPJ, 28% in MCPJ and 5% in both MCPJ and PIPJ. The recurrence rate at an average follow-up period of 61.4 months was 23% (33/143). Seven out of 97 patients with only MCPJ experienced recurrence. On the other hand, out of the 46 patients who had correction at both MCPJ and PIPJ, 26 had recurrence with all at PIPJ and two with associated MCPJ recurrence. In the recurrence group, only five patients required intervention in the form of limited fasciectomy. All patients who underwent surgery for recurrence had a family history of disease. Around 85.1% of patients reported QuickDASH score 0-25, 8.7% reported a score of 25 - 50 and 7.2% reported a score of >50 indicating poor outcome. About 92% of patients were very satisfied with the outcome (Table 3).

**Table 3:** Results Results showing the extent of initial deformity, correction obtained, number of patients that achieved full correction, average follow-up, recurrence rates and patient-reported outcome measures (PROMS) including the number of patients that were very satisfied with the outcome. MCPJ: metacarpophalangeal joints; PIPJ: proximal interphalangeal joint

Parameter	
Initial deformity average, ° (range)	43.6 (28.5 – 74.5)
MCPJ, ° (range)	36.5 (28.5 – 74.5)
PIPJ, ° (range)	48.2 (30.6 – 46.1)
Correction average, ° (range)	4.6 (0 – 25.6)
MCPJ, ° (range)	3.7 (0 – 15.5)
PIPJ, ° (range)	8.7 (0 – 19.8)
Full correction, n(%)	
MCPJ, n(%)	126/143 (88.1%)
PIPJ, n(%)	35/46 (76%)
Average follow-up, months (range)	61.4 (42 – 73)
Recurrence, n(%)	33/143 (23%)
MCPJ, n(%)	7/143 (4.8%)
PIPJ, n(%)	8/46 (17.3%)
PROMS (QuickDASH score)	
Average score (range)	18.5 (0 – 75)
0 – 25, n(%)	121/143 (84.6%)
26 - 50, n(%)	12/143 (8.3%)
>50, n(%)	10/143 (6.9%)
Very satisfied, n%)	132/143 (92.3%)

## Discussion

The study of the digestive activity of filtrates from *Clostridium histolyticum* on equine Achilles was reported in the 1930s. In the late 1990s, its potential therapeutic use in Dupuytren’s disease was reported [8]. Commercial CCH available today comprise a fixed-ratio mixture of two purified collagenolytic enzymes, Clostridial type I collagenase (AUX-I) and Clostridial type II collagenase (AUX-II) [9]. Both have different cleavage sites and catalytic activity and therefore increase enzymatic activity when combined together. CORD I (Collagenase Option for Reduction of Dupuytren) and CORD II were pre-licencing phase III trials that confirmed superior outcome following CCH injection compared to placebo in reducing the Dupuytren’s deformity to <5 ° [10,11]. Similarly, JOINT I and II were open-label studies that reported better outcomes with CCH injection in Dupuytren’s deformity [12].

Multiple studies have shown benefits of CCH in the correction of Dupuytren’s deformity [13-17]. The extent and rate of correction reported in the literature is highly variable but overall correction at MCPJ was better than PIPJ across most of them [10,18]. Correction of 88% at MCPJ and 76% at PIPJ in this study is better than studies with two-year follow-up which showed 80% resolution of MCP deformities and half in PIPJ deformities [19,20].

Local complications of swelling and bruising are very common and usually settle in a few days [21]. In our study, all patients had local complications without any long-term effects. Rare major complications in the form of deep tendon injury, pulley rupture, complex regional pain syndrome, sensory abnormalities, haemorrhage after the procedure and deep vein thrombosis have been reported in the literature [11,20,23]. None of the patients in this series reported any major complications.

CORDLESS (CORD Long-Term Evaluation of Safety Study) was a follow-up study of the patients from CORD and JOINT studies [24]. Recurrence was defined as deformity of > 20 ° in a previously corrected finger. At three years' follow up 35% recurrence was observed with intervention in 8%, while at five-year recurrence at MCPJ and PIPJ was 40 and 66% respectively. Very few studies in the literature have reported long-term outcomes. Watt et al. reported recurrence in 75% of cases at eight years' follow up but the series included only eight patients [25]. The rate of recurrence in this study at an average follow-up of 61 months is 23% with 56% at PIPJ. These results are better than those reported in the literature and we believe that this is due to adherence to strict inclusion criteria [24,25]. All patients were examined for suitability of CCH injection by a senior author and only those patients with a well-palpable cord in one or two digits with deformity >30° at the metacarpophalangeal joint and 20° at the proximal interphalangeal joint were included in the study. Those not suitable for CCH injection were offered surgical intervention.

Multiple studies have studied patient-reported outcomes with the majority reporting good outcomes in >80% [24-27]. QuickDASH score used in this study reported good outcomes in >90% of patients and 92% were very satisfied with the procedure [28].

We appreciate the limitations of this study. Firstly, this is a single-centre and single-surgeon series. This theoretically may have introduced an unconscious selection bias but we do believe that the clearly defined and objective inclusion criteria helped to reduce this significantly. Besides, the criteria for residual deformity or recurrence were similar to those used in the literature. Being a single-centre study, all cases could be monitored in greater detail and heterogeneity of multiple clinicians was avoided. Secondly, 67% of the patients had isolated MCPJ involvement. It could be argued that this may have contributed to better outcomes but we believe that PIPJ deformities are less associated with a distinct palpable cord and therefore were more likely to be excluded from the study.

## Conclusions

To conclude, this study provides good long-term results and patient-reported outcomes of CCH injection in the correction of Dupuytren’s deformity. The local side effects are common without long-term effects and serious complications are rare. The results also indicate that in a carefully selected cohort with a well-palpable cord, CCH is a safe and very effective minimally invasive alternative in the correction of Dupuytren’s deformity with good patient satisfaction rates.
